# Influence of radiation treatment technique (IMRT vs. 3D-RT) on acute toxicity and prognostic factors for survival for anal cancer

**DOI:** 10.1038/s41598-022-24362-8

**Published:** 2022-11-19

**Authors:** Christina Sauter, Jan C. Peeken, Kai Borm, Christian D. Diehl, Stefan Münch, Stephanie E. Combs, Hendrik Dapper

**Affiliations:** 1 Department of Radiation Oncology, Klinikum rechts der Isar, TU München, Ismaninger Str. 22, 81675 München, Germany; 2 Deutsches Konsortium für Translationale Krebsforschung (DKTK), Partner Site Munich, Munich, Germany; 3 Institute for Radiation Medicine (IRM), Helmholtz Zentrum München, Ingolstädter Landstr.1, Neuherberg, Germany; 4 Department of Radiotherapy and Radiation Oncology, Public Hospital of Bielefeld, University Medical Center East Westphalia-Lippe, Bielefeld, Germany

**Keywords:** Cancer therapy, Gastrointestinal cancer

## Abstract

We compared our institutional experience with intensity-modulated radiotherapy (IMRT) and 3D-conformal radiotherapy (3D-RT) for definitive treatment of primary anal cancer. We performed a single-institution retrospective review of all patients with anal squamous cell carcinoma treated with definitive (chemo) radiotherapy with curative intent from 2004 through 2018. We assessed several prognostic factors in respect to relevant survival endpoints. In addition, acute toxicities were determined and compared between IMRT and 3D-RT patients. This study included 94 patients (58 IMRT, 36 3D-RT). Mean follow up for all patients, for IMRT and 3D-RT patients was 61 months (range 6–176), 46 months (range 6–118), and 85 months (range 6–176), respectively. 5-year overall survival (OS) was 86%, disease-free survival (DFS) was 72%, and colostomy-free survival (CFS) was 75% in the IMRT cohort. In the 3D-RT cohort, OS was 87%, DFS was 71%, and CFS was 81% (all *p* > 0.05). Male gender and Karnofsky Index (KI) were revealed as independent prognostic factors for 5-year OS (*p* = 0.017; *p* = 0.023). UICC stage was an independent prognostic factor for DFS and CFS (*p* = 0.023; *p* = 0.042). In addition, the pre-treatment leukocyte count was an independent prognostic factor for CFS (*p* = 0.042). Acute grade ≥ 3 toxicity was not significantly different between IMRT and 3D-RT patients, but the IMRT cohort had favorable outcomes. This study confirmed IMRT as the primary definitive treatment of anal cancer. With similar survival rates, IMRT had the potential to reduce acute toxicity by sparing organs at risk. Promising prognostic factors such as BMI, KI, and leucocyte and hemoglobin levels should be further investigated.

## Introduction

Concurrent radiation with chemotherapy is considered the main therapy for treating non-metastatic anal cancer. The combination of mitomycin C (MMC) and 5-fluorouracil (5-FU) as a chemotherapeutic agent is widely used and accepted^1,2^. According to the National Cancer Comprehensive Network (NCCN), a radiation dose of at least 45 Gy (Gy) in fractions of 1.8 Gy is recommended. A boost of 9 to 14 Gy should be added for more advanced tumors and involved lymph nodes^2^. The 5-year OS in non-metastatic anal cancer ranges from 75 to 79%^3–5^, at T1/T2 stage it is 80% to 90%, and at T4 stage it is 50%^6^. Patients without lymph node involvement at primary diagnosis have a higher 5-year OS than patients with lymph node metastases (63% vs. 37%)^7^. In elderly patients, the diagnosis is more often made at an advanced tumor stage, making age an indirect prognostic factor^8^.

Several acute adverse events caused by concurrent radiation and chemotherapy are important because of their impact on quality of life and continuity of therapy. Therefore, they are often used as the primary or secondary endpoint for treatment evaluation^3,4,9,10^. These include primarily gastrointestinal or genitourinary symptoms^11–13^ and a decline in sexual function^14–16^. Furthermore, radiation can lead to severe radiogenic dermatitis^17^. These radiogenic complications usually occur acutely within hours to weeks after the start of radiation and usually regenerate about two to four weeks after therapy^17,18^. Other patient-dependent factors previously described as prognostic factors in colorectal cancer were the body mass index (BMI) and the Karnofsky Performance Status Scale (KI)^19–21^. Several studies also identified pre-treatment leukocytosis or low hemoglobin count as negative prognostic factors^22–25^.

IMRT became the standard of care for squamous cell carcinoma of the anal canal at our institution in 2010. There are still few comparative data between IMRT and 3D-RT in terms of acute toxicity and survival, so we retrospectively evaluated the outcome at our institution.

## Patients and methods

Patients with histologically proven invasive carcinoma of the anal canal treated with curative radiotherapy (RT) with or without concomitant chemotherapy, either 3D-RT or IMRT, between 2004 and 2018 were included. Patients who received primary surgical treatment or for whom palliative treatment was intended were excluded. All patients underwent pretreatment staging which included digital examination, rectoscopy, MRI or CT scan of the pelvis, and CT of the chest and abdomen. In addition, almost half of the patients received either positron emission tomography (PET) -CT or -MRI. Lymph node metastases were defined as such when the short axis diameter of the lymph nodes was ≥ 1 cm or the morphology of the lymph nodes was suspect (e.g. rounding). The final determination was the responsibility of the specialist in radiology.

For TNM classification, the International Union Against Cancer (UICC) classification system was used^26^*.* All experimental protocols were approved by an Ethikkommission der Technischen Universität München.

After obtaining informed consent from all participants, the results were retrospectively analyzed by reviewing medical records and interviewing patients from October 2018 to March 2019. The documentation of the patient data was performed with the software IS-H (SAP, Walldorf, Germany) und Eclipse 13.0 Treatment Planning System (Varian Medical Systems, Palo Alto, CA, USA).

Patient and treatment specific data, such as age, gender and treatment strategy, BMI and KI were assessed. In addition to the physical condition of the patients, tumor-specific data were also examined. These were the date of diagnosis, complete staging parameters and staging according to the TNM and UICC classifications. Furthermore, laboratory parameters such as pre-treatment leucocyte and hemoglobin levels were assessed as possible prognostic parameters. Human papilloma virus (HPV) and human immunodeficiency virus (HIV) status were not routinely verified for meaningful evaluation.

Particular attention was paid to comparing the efficacy and side effects of the two different radiation techniques IMRT and 3D-RT. We also assessed acute toxicity in patients and its dependence on various patient- and treatment-related factors.

### Radiotherapy

All patients underwent primary radiation with or without concomitant chemotherapy.

A total of 58 patients were treated with IMRT between 2008 and 2018 using either Volumetric Arc radiotherapy (VMAT) or TomoTherapy. Varian Clinac® DHX linear accelerator (Varian Medical Systems, Palo Alto, CA, USA) using image guidance (IGRT) with kilo-voltage cone-beam-CT scans was used for VMAT. Patients regularly received 3 arcs in the main plan and 2 arcs for each boost plan (6 or 15 MV). Hi-ART-System (6 MV) (Accuray, Sunnyvale, USA) including mega-voltage IGRT was used for TomoTherapy. The Treatment Planning System used was Eclipse 13 (Varian Medical Systems, Palo Alto, CA, USA) for contouring and dose comparison. Contouring was performed on planning CTs with 3 mm slice thickness, considering MRI imaging and rectoscopy findings for each patient.

The 36 patients in the 3D-RT group underwent treatment between 2004 and 2008. All patients were treated with Digital Medical Linear Accelerator from Siemens ONCOR™. Planning was performed using Oncentra MasterPlan software Version 3.0 SP1. Contouring was performed on planning CTs with 5 mm slice thickness, taking into account MRI imaging and rectoscopy findings for each patient.

The macroscopic primary tumor (GTVp) and lymph node metastases (GTVn) were defined for all patients using the planning MRI (T2) on the planning CT. A CTV margin of 0.5–1 cm to the CTVp or CTVn was created, taking anatomical limits like bones into account. In addition, the elective lymphatic drainage pathways of the inguinal region (CTVing) and the pelvis (CTVpel) were created in accordance with the contouring guidelines of the British National Guidance^27^. The respective PTVs were then created by a margin of 1 cm of the respective CTVs. Depending on the radio-oncological concept and the risk constellation, the dose was then prescribed on the median volume of the various PTVs.

### Chemotherapy

The majority of patients received 2 cycles of concurrent 5-fluorouracil-based cytotoxic chemotherapy in combination with Mitomycin C. 5-FU was applicated as a continuous infusion (1000 mg/m^2^ per day) on days 1 to 4 and 29 to 32. MMC was applied as an intravenous bolus on day 1 and day 29 of radiotherapy (10 mg/m^2^, maximum 20 mg). Some patients received oral capecitabine (825 mg/m^2^) twice daily on each day of radiation therapy instead of 5-FU during radiation^28^.

### Acute toxicity

Acute toxicity was defined as an adverse event reported between the start of radiation and three months after. Acute toxicity, including diarrhea, dermatitis, genitourinary symptoms, and fatigue was documented in the medical records and classified according to the Common Terminology Criteria for Adverse Events (CTCAE) Version 4.03 (German Version 05/2016, DKFZ Heidelberg)^29^. The severity of adverse events was graded from 1 (mild) to 5 (death due to an adverse event)^29^. In addition, acute toxicity was examined in terms of its correlation with gender, dose, and radiation technique.

### Statistical analysis

All statistical analyses were performed using Statistical Package for Social Sciences software, version 25.0 (SPSS, Chicago IL). Graphics and tables were created using GraphPad Prism, version 8.1.2 (GraphPad Software, San Diego, CA) and Microsoft Office Word, version 16.15 (Redmond, WA).

Chi Square test, Mann–Whitney-U test, and t-test were used for comparison between two groups according to the type of variables. Kaplan–Meier analysis was performed to evaluate the end points of overall survival, disease-free survival, and colostomy-free survival. Each endpoint was calculated from the date of biopsy. DFS was defined as time to tumor recurrence, second malignancy, or death. CFS was defined as the interval to colostomy or death. Variables were tested again by a Cox proportional model. Variables with a *p*-value ≤ 0.2 were included in multivariate analysis. The cut-off for pre-treatment leucocyte count and Karnofsky-Performance Index was calculated by ROC analysis.

Mean values were specified with standard deviation (SD). A two-sided p-value of < 0.05 was considered statistically significant.

### Ethics approval and consent to participate

The study was performed in accordance with the ethics standards at the Technical University of Munich (TUM) (ethical vote: 385/18 s). Name of committee: Ethikkommission der Technischen Universität München.

## Results

### Patient factors and tumor characteristics

Of 94 patients, 58 (61.7%) received IMRT and 36 (38.3%) received 3D-RT. There were 62 women and 32 men with an average age of 60 years (range 38–87). The median follow-up was 50 months (range 6–176). 52 patients had T1 or T2 lesions, 38 patients had T3 or T4 stage; 44 patients had no regional lymph node metastases; 42 patients had N + stage; lymph node status could not be investigated in 8 patients (8.5%). 6 patients (6.4%) had inguinal lymph node metastases solely, 16 (17.0%) had pelvic lymph node metastases solely, and 20 (21.3%) had both inguinal and pelvic lymph node metastases. 41 patients could be classified as UICC stage I or II, 42 were stage III, and 7 patients had distant metastases and were therefore classified as stage IV but treated with curative intent due to an oligometastatic state. UICC status could not be examined in 4 patients.

### Chemotherapy

10 patients (10.6%) did not receive chemotherapy due to small tumor size or multimorbidity. In 6 other patients important information regarding chemotherapy regimen was insufficient. The majority of patients (73.8%) underwent chemotherapy consisting of 5-FU and MMC. 4 of these patients received only one cycle of chemotherapy because they suffered from severe side effects such as dihydropyrimidine dehydrogenase deficiency, deterioration of general condition, intolerable increase in liver enzymes, and abdominal pain.

4 other patients received both cycles with a maximum delay of one week. 3 of them suffered from leucopenia, another 1 was resuscitated due to ventricular fibrillation. 13 patients were treated with oral doses of capecitabine (825 mg/m^2^) twice daily in combination with MMC during radiation on each day of radiation therapy. This combination was newly introduced for the treatment of anal cancer after satisfactory results in the treatment of rectal cancer and the promising results of the phase-II study of Oliveira et al.^30,31^. 3 of these patients had to stop chemotherapy due to severe leucopenia, hand-foot syndrome, and deterioration of general condition. In 1 patient, chemotherapy had to be postponed due to leucopenia.

### Radiation treatment

The median dose applied to the primary tumor was 55.8 Gy (range 50.4–61.2) and the median dose to the involved lymph nodes was 50.4 Gy (range 39.6–59.4) (Table 1).

**Table 1:** Patient characteristics.

Patient characteristics (n = 94)	N (%)		
	Total	3D 36 (38.3)	IMRT 58 (61.7)
**Age at time of diagnosis (years)**			
Median Range	60.1 38–87	58.7 38–85	60.5 41–87
**Gender**			
Female Male	62 (66) 32 (34)	23 (37) 13 (41)	39 (63) 19 (59)
**Follow-up**			
Median Range	50.0 6–176	65.5 6–176	39.0 6–118
**Tumor localisation**			
Anal canal Anal margin	85 (90.4) 9 (9.6)	33 (92) 3 (8)	52 (90) 6 (10)
**Grading**			
G1/2 G3 Gx	51 (54.3) 35 (37.2) 8 (8.5)	21 (58) 13 (36) 2 (6)	30 (52) 22 (38) 6 (10)
**T stage**			
T1 T2 T3 T4 Tx	11 (11.7) 41 (43.6) 22 (23.4) 16 (17.0) 4 (4.3)	2 (6) 16 (44) 10 (28) 6 (17) 2 (6)	9 (16) 25 (43) 12 (21) 10 (17) 2 (3)
**N stage**			
N0 N + Nx	44 (46.8) 42 (44.7) 8 (8.5)	22 (61) 10 (28) 4 (11)	22 (38) 32 (55) 4 (7)
**UICC stage**			
I II III IV x	10 (10.6) 31 (33.0) 42 (44.7) 7 (7.4) 4 (4.3)	3 (8) 18 (50) 12 (33) 2 (6) 1 (3)	7 (12) 13 (22) 30 (52) 5 (9) 3 (5)
**RT dose primary**			
Median Range	55.8 50.4–61.2	55.8 50.4–61.2	58,8 50.4–60
**RT dose involved lymph nodes**			
Median Range	50.4 39.6–59.4	45.0 39.6–59.4	50.4 (45–59.4)
**RT dose elective lymph nodes**			
Median Range	45.0 30.6–50.4	39.6 30.6–45.0	45.0 30.6–50.4
**Chemotherapy**			
MMC/5-FU MMC/Capecitabine Insufficient information regarding protocol Only radiotherapy	65 (73.8) 13 (14.8) 6 (6.4) 10 (11.4)	23 (64) 3 (8) 5 (14) 5 (14)	42 (72) 10 (17) 1 (2) 5 (9)

### Survival outcomes

The median and mean follow-up of our patients was 50 and 61 months (range: 6–176), respectively. The median and mean follow-up after 3D-RT was 66 and 85 months (range: 6–176), respectively. For IMRT patients, the median and mean follow-up was 39 and 46 months (range: 6–118), respectively. 12 patients (13%) died within the first 5 years of diagnosis and 6 others within 10 years, while two-thirds died from the direct effects of anal cancer and one-third from other or unknown causes. 3-year OS of all patients was 89%, 5-year OS was 86%, and 10-year OS was 68% with a total of 18 deaths. 84 of the 94 patients treated (89%) had complete clinical remission. The remaining 10 patients did not have complete remission after 6 months. These patients had a 5-year OS of 80%. Salvage surgery was successfully performed on these patients. In comparison to patients with complete remission, 5-year OS did not differ significantly (*p* = 0.302).

In the entire cohort, DFS was 91% after 1 year, 72% after 3 years, and 71% after 5 years. 19 patients suffered either local tumor recurrence, regional recurrence or distant metastasis at the time of surveillance. 9 of these patients had combined recurrence and distant metastasis.

Of the 12 patients with local tumor recurrence, 2 also had regional lymph node metastases, and 2 others also had distant metastases.

In most of the 10 patients with locoregional lymph node recurrence, there was at least 1 other site where the tumor recurred (5 had also distant metastases, 2 had also a local recurrence). Only 3 patients had just locoregional lymph node recurrence and were treated with re-radiation. Distant metastasis occurred in total in 15 patients within the surveillance period. Seven of them were synchronous or metachronous with other sites of tumor recurrence. The other 8 patients were treated with radiotherapy, chemotherapy, or surgical intervention depending on the site of metastasis. Of the 9 patients with multiple tumor recurrences, none had a survival of more than 2 years. 7 patients died within the surveillance period, another was unreachable after 2 months. Another patient lived until 6 months after tumor recurrence (Table 2).

**Table 2:** Overall survival depending on remission status and metastasis.

	overall (n = 94)		3 years		5 years		10 years	
	n	%	n	survival	n	survival	n	survival
Incomplete remission	10	10.6	7	80%	4	80%	1	80%
Local recurrence	8	8.5	7	100%	6	100%	3	83.3%
Distant recurrence	8	8.5	5	75.0%	4	75.0%	1	24.2%
Combined recurrence	9	9.6	3	51.9%	1	16.8%	0	–

n = patients at risk; combined recurrence = simultaneous locoregional and distant recurrence.

**Figure 1:**
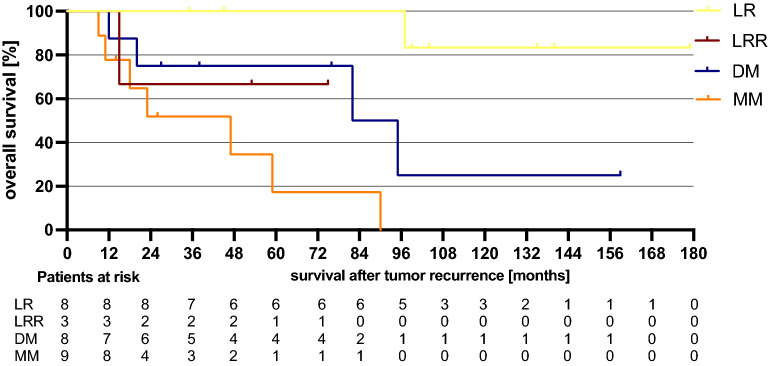
Survival in dependency of remission and recurrence. LR= local recurrence; LRR= locoregional recurrence; DM= distant metastasis; MM=multiple metastases.

Figure 1 shows the overall survival after date of initial diagnosis in dependency of the progress.

Colostomy-free survival was 90% in the first year, 80% after 3 years, and 78% after 5 years. In total, a colostomy was performed in 22% (n = 21). 5 of these patients underwent a protective colostomy prior to therapy. In 4 of these patients, colostomy could be relocated.

Two-thirds of the colostomy-patients (n = 14) underwent salvage surgery, resulting in a colostomy. 2 more patients (10%) received a colostomy in the follow-up period due to gastrointestinal toxicities.

1-year, 3-year, and 5-year OS of the 41 patients with UICC stage I/II was 100%, 97%, and 93%, respectively. The 49 patients with UICC stage III/IV reached a 12-month, 3-year, and 5-year OS of 91%, 81%, and 77%. The differences between the groups were statistically significant (5y-OS *p* = 0.011).

1-year, 3-year, and 5-year DFS was 97.4%, 86.1%, and 82.7%, respectively for stage I/II patients. Patients with UICC stage III/IV reached 88%, 57%, and 57%. Differences in 5-year DFS were statistically significant (*p* = 0.001).

1-year CFS in UICC stage I/II and III/IV was 100% and 79%, respectively. 3-year CFS was 92% and 65%. 5-year CFS was 88% and 65% (*p* = 0.005).

**Figure 2:**
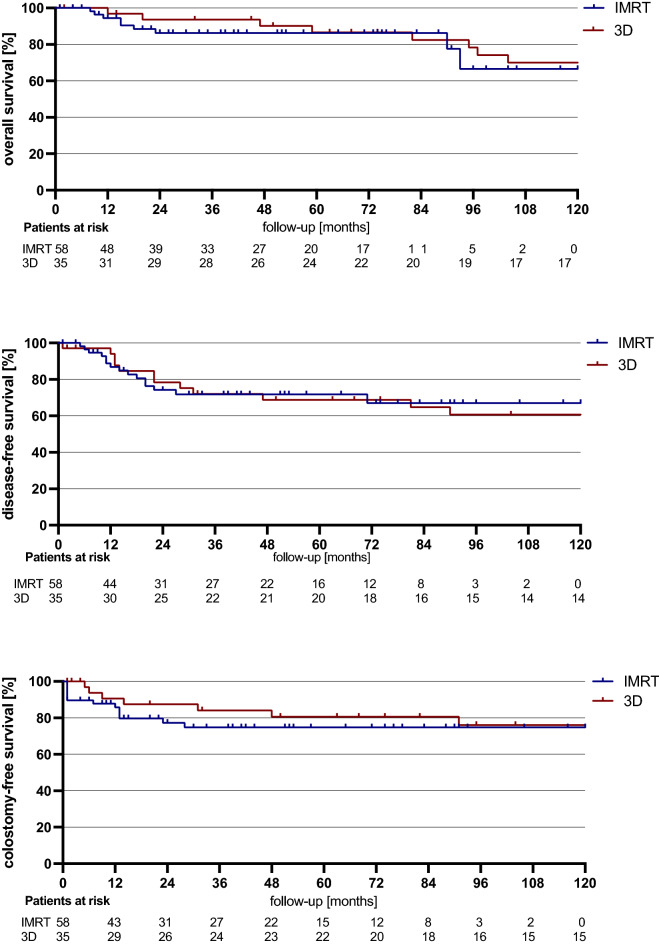
OS, DFS and CFS of IMRT and 3DCRT patients.

Overall survival of 3D-RT patients was 100% at 1 year, 94% at 3 years, and 87% at 5 years. In IMRT patients, the mean 12-month OS was of 95% and the 3-year and 5-year OS were 86% (*p* = 0.607). Disease-free survival of patients treated with 3D-RT at 1, 3, and 5 years was 97%, 74%, and 71%, respectively, whereas the IMRT group reached 87%, 72%, and 72%, respectively (*p* = 0.925). In the 3D-RT cohort, colostomy-free survival was 91% at 1 year, 84% at 3 years, and 81% at 5 years, while the IMRT cohort had a CFS of 86%, 75%, and 75%, respectively (*p* = 0.466) (Fig. 2).

The Karnofsky Performance status scale was used to evaluate functioning in everyday life. In Roc Analysis, a cut-off value of 85 was set to divide the patients into 2 groups. Patients with a KI > 85 had a significantly better 5-year OS, -DFS and -CFS (*p* = 0.020, *p* = 0.028, and *p* = 0.007).

Patients were divided into 2 different groups depending on BMI. The split was made at 25.0 kg/m^2^ according to the S3 guidelines of the German Diabetes Aid^32^.

While the 5-year OS of normal-weight and slightly underweight patients was 93%, the 5-year OS of pre-adipose and obese patients was 69% (*p* = 0.007). The difference in regard to the 5-year DFS was statistically significant as well. Patients with a BMI below 25 kg/m^2^ had a 5-year DFS of 79%, compared to 49% for the pre-adipose and obese patients (*p* = 0.003). Looking at 5-year CFS, there was a tendency for better CFS in normal-weight patients (83% vs. 65%; *p* = 0.098).

Patients with a laboratory hemoglobin value greater than 12 g/dl reached significantly higher 5-year OS (88.3 months vs. 77 months; *p* = 0.034). The 5-year DFS and CFS were not significantly different but tended to be higher in the non-anemic group (5y-DFS: *p* = 0.570; 5y-CFS: *p* = 0.276).

In patients with a blood leucocyte level < 10 G/l, 5-year DFS and 5-year CFS were significantly higher (*p* = 0.022) and (*p* = 0.000). The 5-year OS did not differ significantly but also tended to be higher in the < 10 G/l group (90.2 G/l vs. 72.9; *p* = 0.325) (Table 3).

**Table 3:** KI, BMI, hemoglobin and leucocyte count and clinical endpoints.

	group	n	5y-OS	*p*-value	5y-DFS	*p*-value	5y-CFS	*p*-value
Karnofsky-Index	< 85	18	70.2	**0.020**	51.1	**0.028**	60.0	**0.007**
	≥ 85	58	91.5		77.9		82.5	
BMI	< 25,0 kg/m^2^	60	92.7	**0.007**	78.8	**0.003**	83.1	0.098
	> 25,0 kg/m^2^	21	69.4		48.8		65.1	
Hemoglobin	< 12 g/dl	21	77.0	**0.034**	68.0	0.570	73.0	0.276
	≥ 12 g/dl	58	88.3		72.0		81.3	
Leucocytes	< 10 G/l	68	90.2	0.325	73.8	**0.022**	82.2	**0.000**
	≥ 10 G/l	11	72.9		49.8		48.6	

Kaplan–Meier with log-rank test: *OS* overall survival, *DFS* disease free survival, *CFS* colostomy-free survival. Significant values are in bold.

### Multivariate analysis

Cox regression was performed prior to conducting the multivariate analysis. All variables with a p value of < 0.2 were included in the secondary multivariate analysis.

Multivariate analysis revealed only gender and KI as a significant variable related to OS (hazard ratio (HR)) 5.37; 95% confidence interval (CI), 1.35–21-36; *p* = 0.017; and HR, 0.023; 95% CI, 0.06–0.81; *p* = 0.023. Tumor stage was an independent prognostic factor in DFS HR, 2.53; CI, 1.14–5.64; *p* = 0.023 and in CFS HR, 2.37; CI, 1.03–5.42; *p* = 0.042). For CFS, pre-treatment leucocytes were also found to be a significant independent factor HR, 2.37; CI, 1.03–5.42; *p* = 0.042 (Table 4).

**Table 4:** Uni- and multivariate analysis.

	Cox-regression		Multivariate analysis	
	HR (95% CI)	*P-*value	HR (95% CI)	*P-*value
**5y-OS**				
**UICC**				
I/II III/IV	1 2.40 (1.22–4.73)	**0.011**	1 1.80 (0.75–4.29)	0.186
**Grading**				
G1 G2/3	1 1.20 (0.44—3.25)	0.719	-	
**Gender**				
f m	1 2.67 (1.02–6.94)	**0.045**	1 5.37 (1.35–21.36)	**0.017**
**Radiotherapy**				
IMRT 3D	1 0.96 (0.37–2.53)	0.940	-	
**Karnofsky**				
< 85 ≥ 85	1 0.32 (0.11–0.91)	**0.033**	1 0.22 (0.06–0.81)	**0.023**
**BMI**				
< 25.0 kg/m^2^ ≥ 25.0 kg/m^2^	1 3.56 (1.29–9.83)	**0.014**	1 2.91 (0.84–10.12)	0.111
**Pre-treatment hemoglobin**				
< 12 g/dl ≥ 12 g/dl	1 0.41 (0.14–1.14)	0.088	1 0.50 (0.13–2.00)	0.329
**Pre-treatment leucocytes**				
< 10 G/l ≥ 10 G/l	1 1.84 (0.52–6.51)	0.347	-	
**5y-DFS**				
**UICC**				
I/II III/IV	1 2.66 (1.50–4.73)	**0.001**	1 2.53 (1.14–5.64)	**0.023**
**Grading**				
G1 G2/3	1 1.10 (0.50–2.40)	0.814	-	
**Gender**				
f m	1 2.21 (1.05–4.65)	**0.039**	1 1.62 (0.54–4.89)	0.395
**Radiotherapy**				
IMRT 3D	1 0.88 (0.41–1.85)	0.727	-	
**Karnofsky**				
< 85 ≥ 85	1 0.39 (0.16–0.94)	**0.036**	1 0.53 (0.19–1.50)	0.233
**BMI**				
< 25.0 kg/m^2^ ≥ 25.0 kg/m^2^	1 2.93 (1.28–6.70)	**0.011**	1 2.92 (0.90–9.45)	0.073
**Pre-treatment hemoglobin**				
< 12 g/dl ≥ 12 g/dl	1 0.76 (0.30–1.95)	0.574	-	
**Pre-treatment leukocytes**				
< 10 G/l ≥ 10 G/l	1 2.84 (1.11–7.28)	**0.029**	1 1.68 (0.56–5.07)	0.358
**5y-CFS**				
**UICC**				
I/II III/IV	1 2.34 (1.29–4.25)	**0.005**	1 2.37 (1.03–5.42)	**0.042**
**Grading**				
G1 G2/3	1 1.19 (0.47–2.98)	0.713	-	
**Gender**				
f m	1 1.93 (0.82–4.54)	0.135	1 1.66 (0.50–5.53)	0.410
**Radiotherapy**				
IMRT 3D	1 1.22 (0.50–2.96)	0.662	-	
**Karnofsky**				
< 85 ≥ 85	1 0.30 (0.11–0.80)	**0.017**	1 0.45 (0.14–1.37)	0.159
BMI				
< 25,0 kg/m^2^ ≥ 25.0 kg/m^2^	1 2.20 (0.84- 5.80)	0.109	1 3.11 (0.92–10.52)	0.068
**Pre-treatment hemoglobin**				
< 12 g/dl ≥ 12 g/dl	1 0.63 (0.22–1.81)	0.386	-	
**Pre-treatment leukocytes**				
< 10 G/l ≥ 10 G/l	1 4.95 (1.69–14.52)	**0.004**	1 2.37 (1.03–5.42)	**0.042**

Cox-Regression: 5-year overall survival (OS), disease-free survival (DFS), colostomy-free survival (CFS); *HR* Hazard Ratio; 95% *CI* confidence interval. significant values are in bold.

### Acute adverse events

Besides disorders of laboratory parameters, the most common side effects investigated were radiogenic dermatitis, diarrhea, fatigue, and dysuria.

Only 20 (21.3%) patients did not suffer from dermatologic complications. On average, the tumors in these patients were localized further proximally than those of in patients with radiogenic dermatitis. Grade ≥ 3 radiogenic dermatitis was observed in 26.6%.

There were no significant differences in the incidence of high-grade radiogenic dermatitis in regard to gender or radiation technique (IMRT: 29.3% vs. 3D-RT: 22.2%; *p* = 0.450). However, a significantly lower dose was applied in patients who did not have dermatologic complications (53.8 Gy; SD, 5.9 vs. 56.5 Gy; SD, 3.5; *p* = 0.015).

Radiogenic diarrhea was the second most common complication observed in 62.8% of patients. 12.8% suffered from high-grade diarrhea (CTCAE ≥ 3) during or after therapy. About one-third of the patients suffered from first-grade diarrhea with 2 to 3 stools per day. Second-grade diarrhea with 4 to 6 stools per day was diagnosed in 19.1%. The IMRT treated patients developed diarrhea in 58.6%, while conventionally treated patients developed diarrhea in 69.4% (*p* = 0.291). In regard to the total dose applied, no significant difference was found between patients with (56.1 Gy; SD, 4.8) and without radiogenic diarrhea (55.9 Gy; SD, 3.7) (*p* = 0.287). Looking at gender and radiation technique, there was no significant difference in high-grade radiogenic diarrhea (CTCAE ≥ 3), (*p* = 0.212; *p* = 0.126). A tendency to more frequent high-grade adverse events was noted in 3D-RT patients (IMRT: 8.6% vs. 3D-RT: 19.4%).

Fatigue as a side effect was observed in almost half of the patients. Mild first-grade or second-grade fatigue was noted in 43.6% and 4.3%, respectively. Severe fatigue (CTCAE ≥ 3) was observed in only 1 patient in the 3D-RT cohort. With regard to the cumulative dose of radiation, no significant differences were detected in patients without (55.7 Gy; SD, 4.1) and with fatigue (56.3 Gy; SD, 4.2) (*p* = 0.503). The occurrence of fatigue did not differ significantly between radiation techniques (IMRT: 48% vs. 3D-RT: 50% (*p* = 0.871)). The same applied to the occurrence of third-grade fatigue in gender (*p* = 0.470) and the radiation technique (*p* = 0.565).

**Figure 3:**
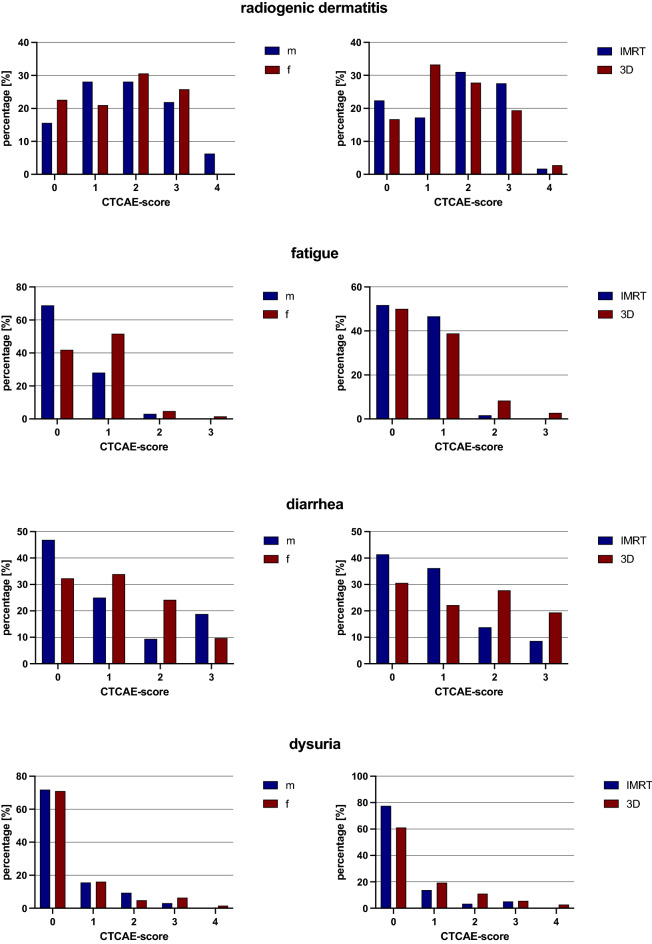
Acute adverse events.

Dysuric problems were reported by about 30% of patients. 16% of patients indicated dysuria equivalent to first-grade dysuria and 6.4% had alguria (CTCAE 2). Dysuria was noted in the conventionally treated patients at a 61% higher rate than in the IMRT group (*p* = 0.125). In terms of mean radiation dose, there were no significant differences in the occurrence of dysuria (dysuria: 55.27 Gy; SD, 3.74; no dysuria: 56.34 Gy; SD, 5.03) (*p* = 0.278). Severe dysuria (CTCAE ≥ 3) was observed in only 6 patients, 3 of whom belonged to the IMRT group (5%) and another 3 to the 3D-RT group (8%) (*p* = 0.542). Gender showed no significant difference when considering high-grade dysuria (CTCAE ≥ 3) (*p* = 0.353) (Fig. 3).

## Discussion

So far, there is insufficient data on the adequate radiation dose prescription for anal cancer, which is why the British Plato study (ACT III-V) is currently investigating the personalized dosing according TNM^33^. Hence, due to different risk factors (TNM), the range of dose prescription to the primary, affected lymph nodes and the elective lymphatic drainage pathways was relatively high in our collective. The large range can also be explained by the discontinuation of therapies. However, the respective median dose to the primary, affected lymph nodes and the elective lymphatic drainage pathways largely corresponds to that of the Plato study^33^.

Five- year OS of our cohort was 85.5%. Lower survival rates of 75% to 79% have been reported in large randomized controlled trials^3–5^. One reason for better OS in our cohort compared to the aforementioned studies could have been consequent tumor surveillance after treatment with modern systemic treatment modalities and improved salvage therapy. Another reason could have been more sensitive pre-treatment staging, which included MRI and also PET-MRI, or CT in 45% of our patients. The role of a more sensitive staging including PET imaging has already been described in a failure analyses in the context of common contouring guidelines by Dapper et al.^34^. More advanced tumor stages or stages of metastasis were potentially underrepresented in the randomized controlled studies. Even at the stage of oligometastasis, patients benefited from minimally invasive methods such as stereotactic ablative radiotherapy. With ACT II (MMC/5-FU) and 3D-RT, a 5-year DFS of 69% was observed^3^. In our cohort, 5-year DFS was 71%, similar to previous studies. This suggests that both techniques are similarly efficient in terms of survival. The randomized controlled trials ACT II, RTOG 98–11, and Accord 03 described a 5-year CFS of 68% to 77%^3,4,35^. The upper range is similar to our cohort with a 5y-CFS of 78%. However, an exact comparison is not possible, since definition of CFS varied between the studies. In Accord 03 and ACT II, protective colostomy was not included in CFS^3,35^.

Gender was an independent prognostic factor regarding OS. This is congruent to other studies, where gender is described as prognostic factor as well^7,36^. It can be assumed, that women generally tend to become older, and women with anal cancer have a higher prevalence of HPV infections^37^. The reason remains unclear though and might be multifactorial, since the DFS didn’t differ significantly.

In a review of Das et al., tumor size and lymph node status are described as the most important and reliable prognostic factors^38^. Patients from RTOG 98–11 who had T3/4 N + status had the worst prognosis^4^. Patients with UICC ≥ III of our cohort also had a significantly greater risk in relation to 5-year OS, DFS and CFS compared with patients with UICC I/II. This underlines the importance of sensitive staging, e.g., by PET-CT, before starting therapy.

In our study, there was no significant difference in 5-year OS, DFS and CFS regarding both radiation techniques. Chuong et al. also described similar survival rates in patients treated with IMRT and 3D-RT (3y-OS IMRT 86%; 3y-OS 3D-RT 92%)^39^. Other authors confirm these findings, stating that there is no difference in survival endpoints concerning conformal radiotherapy and IMRT^40,41^. Generally, target volume definition, dose prescription, and PTV coverage are identical when using 3D-RT and IMRT technique, but dose sparing of organs at risk in high dose range can be better implemented with IMRT. Kachnic et al. examined the contouring of patients in the MMC/5-FU arm of RTOG 98–11. They described similar doses in GTV and CTV using 3D-CRT and IMRT contouring techniques, but toxicity of the latter is lower^42^. Ultimately, similar tumor control can be expected with both radiation techniques and the better tolerability of IMRT (see RTOG 0529) does not have a positive effect on colostomy clearance at least in small cohorts. Due to the unequal follow-up period (mean IMRT: 39 (range, 6–118) months, and mean 3D-CRT: 65.5 (range, 6–176) months) there might be a background bias between the two cohorts, that might potentially affect the clinical endpoints. Age at time of diagnosis and radiation doses remained similar, though.

Not surprisingly, a higher KI showed a tendency to a higher OS in studies of rectal- and colon cancer^19,20^. Our anal cancer cohort also reached significantly higher 5-year OS, DFS and CFS with a KI > 85 (*p* = 0.020; *p* = 0.028; *p* = 0.007). In addition to an increased number of comorbidities, advanced age also played a role in patients with lower KI. The median age of patients with a KI < 85 was 65 years, whereas the median age of patients with a KI > 85 was years. In a study by the National Cancer Data Base, age > 65 years was described as a negative prognostic factor in anal cancer patients^7^. Elderly patients usually have a more advanced tumor stage, so age can be considered an indirect prognostic factor^8^. In a review by Nilsson et al., age > 70 years was even described as an independent prognostic factor for overall survival (HR: 2.86, CI 1.88–4.34). Another reason for the worse results in patients with lower KI was the smaller proportion of patients who received chemotherapy. 28% did not undergo an adjuvant therapy because of multimorbidity. It was only 6.6% in the group of patients with KI > 95.

Adipose patients had a significantly lower 5-year OS and DFS than normal-weight patients (*p* = 0.007; *p* = 0.003). As a component of the metabolic syndrome, it is a major cardiovascular risk factor associated with high morbidity and other diseases^43,44^.

An analysis of ACCORD 03 and KANAL 2 by Faivre et al. showed a potentially higher risk of colostomy in patients with a BMI of 25–30 kg/m^2^ (*p* = 0.170). This could not be confirmed in our study due to a smaller cohort. Both metabolic syndrome and radiation contribute to microangiopathy. The release of oxidative stress and leakage of free radicals induce apoptosis and damage in the involved vessels^45^. This results in radiogenic complications such as ischemia, perforations, bleeding, and wound healing disorders.

However, other authors describe a paradox regarding survival of obese cancer patients. A review by Lennon et al. reported a higher survival rate in patients with an increased BMI compared to normal-weight patients^46^. An excess of adipocyte tissue as an endogenous reserve for stress is discussed as a hypothesis for the survival advantage^46^.

Other explanations for this paradox could be methodic failures, a less aggressive tumor biology, or a better response to therapy^46^. Nevertheless, BMI has been recognized as a predictive factor for radiation induced hematologic toxicity in anal cancer and prostate cancer patients^47,48^ . Mell et al. describe women with low BMI and node-positive disease would most benefit from bone-marrow-sparring IMRT, as decreasing BMI was significantly associated with lower white blood cell count^47^. Therefore, an elevated BMI could be protecting with regard to hematologic toxicities and resulting complications. In addition, it is discussed that BMI is inappropriate as a measurement system depending on the general body constitution, thus it is not an independent risk factor for survival^46^. This goes together with the findings in our cohort, where high BMI was not shown to be an independent risk factor in multivariate analysis. The extent to which BMI can be used as an independent risk factor and pre-obesity in particular might be protective is not entirely certain and should be evaluated in prospective studies. Finally, moderate obesity tends to have a positive effect, while massive obesity has a detrimental effect.

Previous results proved the influence of pre-treatment hemoglobin levels on the prognosis for patients with anal cancer^22^. The correlation between hemoglobin level and tumor oxygenation has been described in several types of cancer, especially squamous cell carcinoma of the head and neck^49,50^. Sufficient oxygenation effectuated a better response to radiation and chemotherapy compared to a hypoxic state^51–53^. Tumors were more hypoxic than the surrounding tissues and hypoxia was considered one of the most important factors in tumor resistance to radiotherapy^53^. During the anemic state, the proportion of hypoxic tumor cells could increase, reducing the success of therapy^23^. This effect was also reflected in our cohort. The 5-year OS of anemic patients was significantly lower than of patients with pre-treatment hemoglobin levels > 12 g/dl (*p* = 0.034). Hemoglobin levels could not be confirmed as an independent prognostic factor for the 5-year OS (*p* = 0.329). 81% of the patients with pre-treatment anemia reached UICC stage ≥ 3. Because of their size, more advanced tumors tend to bleed, which may lead to a relevant decrease in hemoglobin level. This is reflected in the results of EORTC 22,861 and RTOG 98–11 in which pre-treatment hemoglobin levels were also not defined as prognostic factor^10,54^. However, in a retrospective study by Franco et al. with 161 patients included, significantly higher 5-year PFS and -OS were observed in patients with pre-treatment hemoglobin levels > 12 g/dl^55^. We cannot rule out the possibility that diseases other than anal cancer had an impact on hemoglobin levels. Nonetheless, the value of hemoglobin seems to be promising as a predictive parameter and should be investigated in prospective long-term studies, as is already known for other pelvic tumors such as cervical carcinoma^56^.

Another blood parameter that has been established in the literature but not yet in clinical practice is the pre-treatment leucocyte level. Several retrospective analyses as well as ACT I could show a correlation between pre-treatment leukocytosis and worse treatment outcome^22,25,57^. Another promising laboratory parameter that was previously discussed was the neutrophil–lymphocyte ratio^58^. Neutrophil count was not standardly included in the laboratory test, therefore we focused on the role of leucocytes. The role of leucocytes as a predictive parameter must be explained by their function as inflammatory parameters.

Moreover, radiation-induced hematologic toxicity, and particularly radiation-induced lymphopenia has been identified as a prognostic marker in previous studies. Radiation of the bone marrow- as part of the lymphatic organ- has been associated with clinically significant hematologic toxicity^59,60^. The resulting lymphopenia coheres with an increased risk for opportunistic infections and worse oncologic outcomes due to essential role of lymphocytes in the anti-tumor response. Techniques to reduce bone marrow irradiation, as IMRT, could reduce hematologic toxicities in anal cancer patients^47^.

Meanwhile, inflammation as an intrinsic factor is attributed to an important role in tumor genesis and progression^61^. Analogously to the results in literature, patients with a leucocyte count < 10 G/l did not have a significant benefit in 5-year OS (*p* = 0.325). Nevertheless, the risk for recurrent disease or colostomy was significantly higher in patients with a pre-treatment leucocyte count > 10 G/l (*p* = 0.029; *p* = 0.004). Furthermore, the multivariate analysis identified a leukocyte count > 10 G/l as an independent prognostic factor for 5-year CFS (*p* = 0.042). Other factors and diseases could lead to inflammation and thus to increased leucocyte counts. In our cohort, KI as a surrogate parameter for patients’ general condition did not negatively correlate with leucocyte count, underscoring the importance of leukocyte count as an independent risk factor.

### Acute toxicity

Many acute toxicities following concomitant radiation and chemotherapy are described in the literature and are considered important enough to be used as a primary or secondary endpoint for evaluating the tolerability of medical treatment^3,4,9,10^.

The most frequent acute toxicity in our cohort was radiogenic dermatitis in 80% of patients (n = 75). Pathogenesis was complex and a result of a combination of different factors, including the dose applied, dose per fraction, tissue treated, chemotherapy, local infections, and other comorbidities^62,63^.

In our cohort, patients without radiogenic dermatitis had received a significantly lower dose than patients with dermatologic manifestations. Radiotherapy leads to nuclear and mitochondrial damage as well as tissue destruction and the development of reactive oxygen species, which—in combination with stem cell damage—can cause alterations in endothelial cells, inflammation, and necrosis^17^. No differences were found in our patients with respect to the radiation technique. Pronounced radiodermatitis can therefore be severe and lead to therapy interruptions, ultimately worsening the patient’s prognosis. This is another important argument for the widespread use of potentially less toxic radiotherapy techniques such as IMRT.

The second most frequent toxicity was diarrhea in 63% of patients. 9% of the IMRT cohort and 19.4% of the 3D-RT cohort suffered from a high-grade diarrhea (CTCAE ≥ 3) (*p* = 0.126). In RTOG 98–11, third-grade diarrhea occurred in 24% of patients treated with MMC/5-FU and 3D-RT.^4^. Diarrhea was basically caused by incidental radiation of the small bowel^40^. The high dosage area was smaller using IMRT technique. Therefore, RTOG 0529 resumed that no more than 200 cm^3^, 150 cm^3^, 20 cm^3^, and 0 cm^3^ of the small bowel should receive a dose higher than 30 Gy, 35 Gy, 45 Gy, and 50 Gy, respectively^42^. A secondary analysis of the same study revealed a significant correlation between dose applied to the small bowel and increased risk of acute gastrointestinal toxicities ≥ 2 in volumes of 186 cm^3^, 155 cm^3^, 41 cm^3^, and 30 cm^3^ and doses of 25 Gy, 30 Gy, 35 Gy, and 40 Gy, respectively^64^. These data demonstrate that incidental radiation of the small intestine can be reduced with IMRT technique, thereby reducing acute gastrointestinal toxicity. It also underscores the importance of considering various constraints when planning therapy.

Another side effect observed in almost half of our patients was fatigue. Higher-grade fatigue (CTCAE ≥ 3) was observed in just 1 patient of the 3D-RT cohort (3%). Other authors also barely report higher-grade fatigue in their patients^65^. Generally, fatigue is named as the most common side effect of a tumor disease or its therapy^66^. The literature describes the occurrence of fatigue in up to 80% of patients during or shortly after radiotherapy, which is accompanied by loss of appetite, nausea, and vomiting^67^. Despite this high number of affected patients, the symptom of fatigue is rarely sufficiently managed in clinical practice. Only half of the patients get to talk to a physician about this symptom and even less often a therapy is proposed^68^. Even in our cohort, the symptom of fatigue seemed to be underrepresented compared with clinical experience. This illustrates the importance of systematic and all-encompassing follow-up of cancer patients and a consistent range of accompanying therapies.

Nearly 30% of patients reported dysuric symptoms. 5% of our IMRT cohort and 8% of our 3D-RT cohort suffered from a severe dysuria CTCAE ≥ 3. In the randomized controlled trials by James et al. and Ajani et al., these symptoms were reported in 1.3% and 3.4% of patients^3,4^. Only 2% of patients treated with IMRT in RTOG 05–29 showed a higher-grade acute urogenital toxicity^42^. Comparing the two techniques, urogenital tract complications occurred in 5% of patients in the 3D-RT group of Chuong et al., whereas the IMRT group had no higher-grade toxicity^39^. Although not consistently significant, the use of IMRT shows the potential to reduce acute toxicity by sparing organs at risk, and therefore may also alleviate symptoms in some cases, potentially even avoiding treatment interruptions and complications and therefore positively affect patients’ quality of life^69^.

All in all, these results again show that highly conformal techniques such as IMRT have advantages over 3D-RT and should therefore be widely used.

## Conclusion

This study confirmed IMRT as the primary definitive treatment of anal cancer. With similar survival rates, IMRT offers the opportunity to reduce acute toxicity by sparing organs at risk. Promising prognostic factors such as BMI, KI, leucocyte count, and hemoglobin levels should be further evaluated in the future.

## Data Availability

The present data are summarized in this paper ("METHODS" Section). The full data set is available to interested readers upon formal request to the authors.

## Abbreviations

3D-RT: Three-dimensional radiotherapy
5-FU: 5-Fluorouracil
BMI: Body mass index
CFS: Colostomy-free survival
CI: Confidence interval
CT: Computer tomography
CTCAE: Common Terminology Criteria for Adverse Events
DFS: Disease-free survival
Gy: Gray
HIV: Human immunodeficiency virus
HPV: Human papilloma virus
HR: Hazard ratio
IGRT: Image-guided radiotherapy
IMRT: Intensity modulated radiation therapy
KI: Karnofsky Performance status scale
MMC: Mitomycin C
MRI: Magnet resonance imaging
NCCN: National Cancer Comprehensive Network
OS: Overall survival
PET: Positron emission tomography
RT: Radiotherapy
SD: Standard deviation
UICC: Union Internationale Contre le Cancer
VMAT: Volumetric Arc radiotherapy
